# A memristive-photoconductive transduction methodology for accurately nondestructive memory readout

**DOI:** 10.1038/s41377-024-01519-w

**Published:** 2024-07-23

**Authors:** Zhe Zhou, Yueyue Wu, Keyuan Pan, Duoyi Zhu, Zifan Li, Shiqi Yan, Qian Xin, Qiye Wang, Xinkai Qian, Fei Xiu, Wei Huang, Juqing Liu

**Affiliations:** 1 Key Laboratory of Flexible Electronics (KLOFE) & Institute of Advanced Materials (IAM), Nanjing Tech University (NanjingTech), 30 South Puzhu Road, Nanjing, 211816 China; 2 Shandong Technology Center of Nanodevices and Integration, School of Microelectronics, Shandong University, Jinan, 250100 China; 3 Shaanxi Institute of Flexible Electronics (SIFE), Northwestern Polytechnical University (NPU), 127 West Youyi Road, Xi’an, 710072 China

**Keywords:** Photonic devices, Optoelectronic devices and components

## Abstract

Crossbar resistive memory architectures enable high-capacity storage and neuromorphic computing, accurate retrieval of the stored information is a prerequisite during read operation. However, conventional electrical readout normally suffer from complicated process, inaccurate and destructive reading due to crosstalk effect from sneak path current. Here we report a memristive-photoconductive transduction (MPT) methodology for precise and nondestructive readout in a memristive crossbar array. The individual devices present dynamic filament form/fuse for resistance modulation under electric stimulation, which leads to photogenerated carrier transport for tunable photoconductive response under subsequently light pulse stimuli. This coherent signal transduction can be used to directly detect the memorized on/off states stored in each cell, and a prototype 4 * 4 crossbar memories has been constructed and validated for the fidelity of crosstalk-free readout in recall process.

## Abstract (web summary)

A memristive-photoconductive transduction (MPT) behavior was firstly observed in a memristor based on porous P3HT, which is the direct signal for cell resistance states detection, enabling accurate and nondestructive readout.

## Graphical abstract

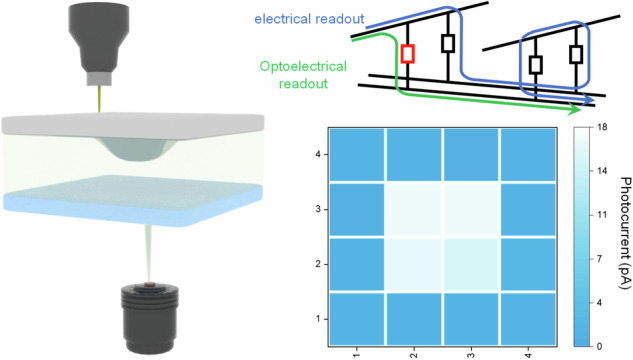

## Introduction

Resistive memory technologies with crossbar architectures have huge potential for high-density information storage^1,2^. Whereas, the undesired sneak current flowing in adjacent memory cells can induce the crosstalk issue, leading to significantly limited array size, increased system dissipation and decreased fault tolerance^3,4^. To mitigate this sneak path, several configurations, including one transistor-one resistor (1T1R), one diode-one resistor (1D1R) and one selector-one resistor (1S1R), have been developed by integrating cell with additional switching or rectifying units^5–11^. Despite avoiding the distortion of data stored in selected cell during a reading operation, these existing approaches considerably complicate circuit design and manufacturing process. Complementary resistive switching is also a significant way that blocks sneak current effectively while suffers from a destructive readout^12,13^. In light of the drawbacks of prevailing strategies, exploiting new method to accurately and nondestructively read memory within 1R-only design is highly demanded.

Recent in-situ microscope and spectral probe studies have revealed the structural and element evolution of resistive memories during the set/reset process, convincingly displaying that metallic particles or oxygen species could diffuse to guide conductive filament formation and fuse, resulting in resistance variation of memristive layer^14–21^. The resulting variations cause a significant impact on their photoelectric behaviors in most semiconductive memories^22–24^. For instance, light emission in an organic diode memory was observed in its high resistance state, yet vanished in low resistance state due to the contiguous metal bridge formed between two terminals, in which carriers recombined along the conductive bridge paths after electrical transition^25^. Meanwhile, photoelectric effect normally exists for photodetection in semiconductive diodes^26–28^. The growth of metallic conductive filaments within memristive medium may bring about changes in the photoelectric features of memory cell, which can be employed as readout signal for binary information recognition.

In this work, we present a reliable memristive-photoconductive transduction (MPT) strategy for precise sensing of the resistance state in a semiconductive memory device. The device comprises an intervening poly(3-hexylthiophene-2,5-diyl) (P3HT) film and two electrodes Ag/ITO, exhibiting bistable resistive switching behaviors under bias and relevant photoresponses under light exposure. The diffusion of silver atoms into P3HT layer produces more trap centers, leading to persistent photoconductance (PPC) that further corroborated by scanning transmission electron microscopy (STEM) and Kelvin probe force microscopy (KPFM) techniques. Upon the metallic conductive filaments connect the two electrodes, which cause the disapperance of photoconductive response and realize MPT behavior. Finally, we fabricated a prototype crossbar array where each crosspoint contains a memory cell with this tunable photoconductive response, and experimentally investigate the switchable optoelectrical signals for nondestructive and crosstalk-free readout.

## Results

**Fig. 1:**
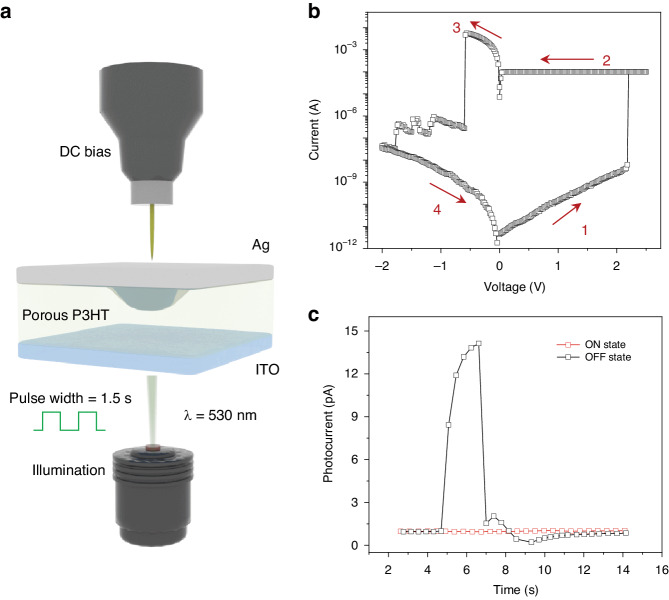
Device configuration and its memristive-photoconductive transduction. **a** Schematic of the prepared memory device with an intervening porous P3HT film sandwiched between top metal and bottom transparent electrodes. **b** The I-V curves pose an integral write-erase cycle (HRS-LRS-HRS) of the device, indicating a typical rewritable nonvolatile memory function, where the numbers and arrows suggest the sweeping sequences. **c** The photoresponse of the device under different resistance states (0 bias). The OFF state device (HRS) exhibits significant photocurrent under illumination, which vanished upon switching to ON state (LRS)

The polymer semiconductor P3HT serves as a prominent material in the field of organic electronics and optoelectronics, such as thermoelectrics, photovoltaic cells, and transistors^29–32^. Furthermore, its high HOMO energy level, wide band gap (~1.9 eV), and visible light absorption make it a promising candidate for photodetectors^33,34^. By constructing fine nanostructures on the surface of P3HT film, controllable resistive switching behaviors are realized as well in which the interface-engineered film as the resistive medium^35,36^. Consequently a memory cell was fabricated through directly spin-coating P3HT layer onto the pre-cleaned ITO bottom electrode, and subsequently deposited conductive silver (Ag) layer as the counter-electrode. Quintessential cell architecture of Ag/P3HT/ITO layer stack is depicted schematically in Fig. 1a. The intermediate P3HT layer was spun-cast in a high humidity, resulting in the formation of nanopores on its surface attributed to the breath-figure-method (Fig. S1a)^37^. The cells exhibited typical resistive switching behavior, which initially maintains a high resistance state (HRS, defined as OFF state) within the bias range of 0 ~ 2.2 V (Fig. 1b, step 1). Then a transition from HRS to low resistance state (LRS, defined as ON state) occurs at 2.2 V. A current compliance of 100 μA was employed to limit the ON state current, which is beneficial to improve cell reliability and uniformity. The cell could preserve the LRS in the absence of external bias, and return to the HRS when the applied bias decreased to -0.6 V, indicating a reset process. Moreover, the cell has good endurance, high resistance ratio (>10^5^), pleasurable retention (>10^4^ s) and uniform bias distribution of both set (~2.8 V)/reset (-0.68 V) operations (Fig. S2a–d).

Meanwhile, the photoconductive measurement was implemented by a pulsed 530 nm illumination with an intensity of 0.52 μW cm^-2^ and a duration of 1.5 s (determined by the absorption spectra of the as-deposited P3HT layer, Fig. S1b). Figure 1c shows a gradually increased photocurrent of a pristine memory cell during the illumination, which quickly falls back the baseline after withdrawing the light. Whereas, the same cell exhibited no significant current fluctuation when it switched from its initial HRS to LRS during an identical test, which is ascribed to the formation of metal conductive filaments in the electroactive matter, thus leading the recombination of charge carriers along the conductive paths (Fig. 1c). A missing photoresponse appears again for the same cell returned to the HRS, yet certain discrepancies in the relaxation time, defined as the decay time from its peak value to the baseline, were observed (Fig. S3). The pristine cell exhibited stable cyclic transient photoresponses with a short relaxation time of ~0.93 s (Fig. S3a, c). This can be ascribed to the efficient separation of light generated excitons within the intervening layer, followed by the migration of separated holes and electrons towards the ITO and Ag ends, respectively owing to the potential difference induced by asymmetric electrode designs^38–40^. After undergoing an integral set-reset process, the same cell still poses stable photoresponse, but the relaxation time is extended to ~4.85 s, indicating a typical PPC phenomenon (Fig. S3b, d)^41–43^. We hypothesize that this is associated with the growth dynamics of conductive filaments within the P3HT layer.

**Fig. 2:**
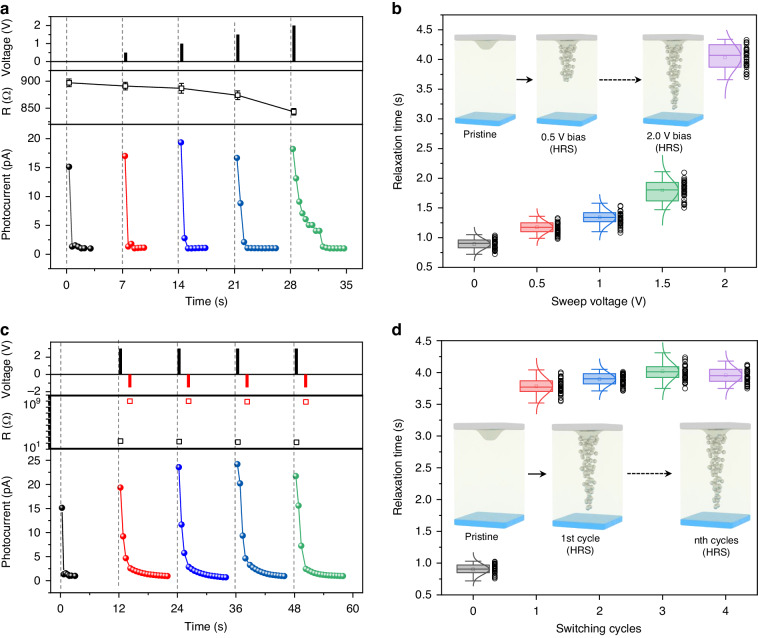
Photoelectric characteristics. **a** The evolution of resistance and photocurrent of a pristine device under five different scanning ranges (0.0, 0.5, 1.0, 1.5, 2.0 V). **b** The distribution of the photocurrent relaxation time for 30 random cells at each bias and the possible structure and element evolution within the P3HT layer (inset images). **c** The evolution of resistance and photocurrent of another device underwent multiple transition (photocurrent data was collected from the HRS device). **d** The distribution of the photocurrent relaxation time for 30 random cells at initial and each write-erase cycle (HRS → LRS → HRS) states. The inset image depicts the proposed structure and element evolution within the P3HT layer

To hinge the experimentally observed signal transduction between the memristor and photoconductivity, the dependence of relaxation time on different bias ranges and switching cycles were investigated. With five successive scans ranging from 0.0, 0.5, 1.0, 1.5, to 2.0 V (Fig. 2a), the cell resistance decreases gradually, without any dramatically changes as the bias increases, but the photocurrent presents with stepwise increase of relaxation time, indicating the maintenance of HRS during the whole scans. The distribution of the photocurrent relaxation time for 30 random cells at each bias was calculated in Fig. 2b. In their initially state, the relaxation time predominantly ranged from 0.72 and 1.05 s with a Gaussian center of 0.81 s, there is no penetration of metallic elements into the semiconductive medium. The time increases significantly in response to the continuously increasing scan bias, suggesting the gradually permeation of metal elements into the medium, which serves as trap centers that effectively capture photogenerated charge carriers (inset of Fig. 2b). A high bias results in the existence of more trap centers within the semiconductor, thereby achieving a longer relaxation time.

Subsequently, another 30 cells were selected randomly to explore the correlation between relaxation time and switching cycles. These cells underwent multiple resistive transitions between HRS and LRS, with their photoresponse features being examined in both the initial state and HRS after each reset operation (Fig. 2c). The distribution of relaxation time for pristine cells primarily ranges from 0.7 to 1.03 s with a Gaussian center of 0.88 s. Subsequent to the first complete transition (HRS → LRS → HRS), there is a significant increase in relaxation time, conforming effective infiltration of metal elements into the switching medium. The relaxation time exhibits minimal fluctuations during the subsequent cycles, demonstrating a dynamic process for reconnecting fused conductive filaments rather than generate new conductive paths (inset of Fig. 2d).

**Fig. 3:**
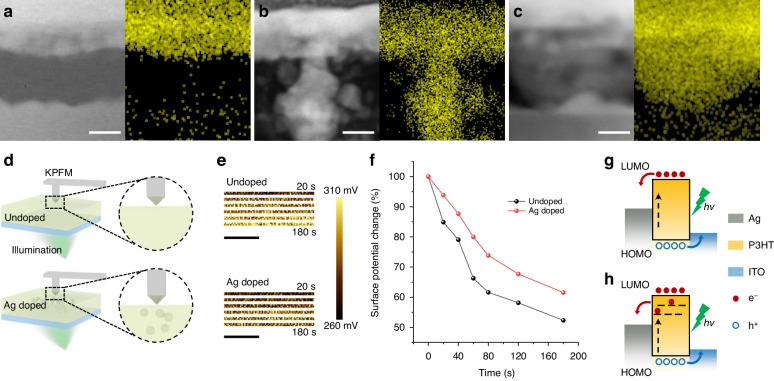
Structural component and charge trapping exploration. The cross-sectional TEM image and Ag element mapping of the memory (**a**) in the pristine HRS, (**b**) in LRS after a set operation and (**c**) in LRS after a reset operation. scale bars: 120 nm. **d** Schematic diagram of the experimental setup for surface potential measurement in KPFM mode. **e** The surface potential evolution of Undoped Ag doped P3HT film after illumination. Scale bars: 300 nm. A small scanning area is employed to ensure real-time performances. **f** The calculated relative surface potential changes of pure P3HT film and Ag doped P3HT film at different time after removing the illumination. **g** and **h** The schematic energy band diagram of pure P3HT film and Ag doped P3HT film. The nanoparticles in P3HT layer act as trap centers that improves the charge trapping ability, resulting in a PPC effect

In order to evidence the above growth kinetics model of conductive filaments, the evolution of microstructure components inside the medium at distinct store states were observed by cross section STEM, and their corresponding elements were identified by the energy dispersive spectrometer (EDS) mapping (Fig. 3a–c). The pristine memory cell showcases a well-defined layered configuration, wherein the P3HT layer isolates the two electrodes completely, thus the cell behaves an insulative state (Fig. 3a). The absence of Ag migration can assist the efficient dissipation of photogenerated carriers for fast response. Figure 3b illustrates the growth of a distinct Ag filamentary bridge across the entire intermedium during a set operation, which is primarily driven by the electrochemical metallization of Ag ion under an electric field^44^. By undergoing a reset, the filament rupture into scattered Ag particles (Fig. 3c), which contribute the insulation and PPC recovery of cell.

We further experimentally investigate the charge trapping capabilities of Ag nanoparticles (AgNP) in a polymer matrix. For this purpose, two types of P3HT thin films with and without Ag particle doping were prepared. Using in situ KPFM test, the pure film (Undoped) exhibits an initial surface potentials of 348 mV, while the composite film (Ag doped) shows a slightly lower potential of 331 mV (Fig. S4a, b). Upon exposure to white light, the two potentials decrease to 262 and 266 mV, respectively, owing to the photogeneration of charge carriers within photoresponsive matters^45^. After removing the light, the temporal evolution of surface potential was monitored by employing a small scanning area (Fig. 3e). The surface potential images of pure and composite films at 20, 40, 60, 80, 120 and 180 s after removing the illumination are arranged in sequences. The surface potential of pure film progressively increases from 262 mV to 303 mV at 0, 20, 40, 60, 80, 120 and 180 s relaxation time, indicating a 47.7% loss of initially trapped charge carriers (Fig. 3f). In contrast, the Ag doped P3HT film poses a similar increasing trend in surface potential over time, but with only 38.5% relaxation of initially trapped charge carriers after 180 s (266, 270, 274, 279, 283, 287 and 291 mV at 0, 20, 40, 60, 80, 120 and 180 s, respectively, Fig. 3e, f). The improved charge retention gives evidence that the Ag infiltration into medium can act as trapping centers, effectively enhancing the capacity of trapping carriers. Therefore, the variations in photoresponse relaxation time of semiconductor memory cell originates from the capture and gradual release of photogenerated carriers by Ag trap centers within the P3HT layer^41^.

**Fig. 4:**
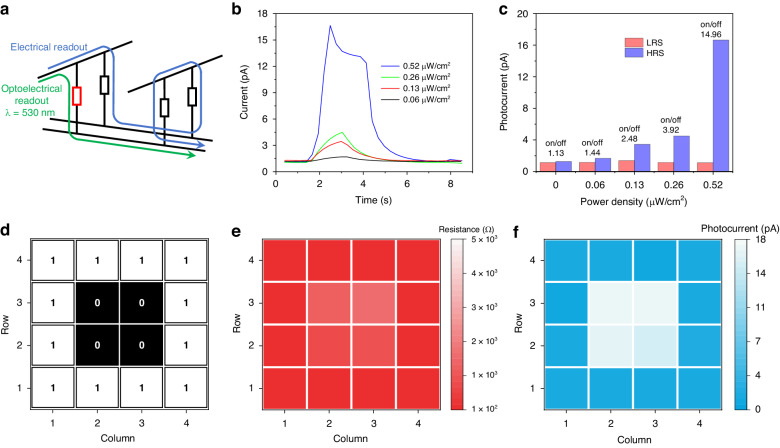
MPT-assisted accurate retrieval. **a** Crosstalk issue in a memristive crossbar configuration. The addressed HRS cell (red) is surrounded by LRS cells (black), which is one possible harsh case pattern. The sneak current flow through the neighboring cells would confuse the periphery, leading to incorrect interpretation of recorded data during the electrical reading. In the same scenario, by illuminating the target cell with green light through the transparent electrode, the memory cell in HRS generates detectable photocurrent, which disappears for its LRS counterparts owing to the direct connection of two terminals (i.e., short-circuited), accordingly the memory states can be sensed via this MPT strategy. **b** The photocurrent curves of the memory cell in HRS at different incident light power densities. **c** The effect of incident power on photocurrent on/off ratio in HRS (on) and LRS (off). **d** A prototype 4 * 4 memory array based on crossbar configuration with preset resistance (black for HRS, white for LRS). **e** Resistances of all 16 cells are identified by electrical readout, which is difficult to distinguish the cells with different resistance states. **f** Photocurrents of all 16 cells are illustrated by optoelectrical readout, which is feasible to identify the memory cells in HRS

Benefitting from the intrinsic MPT capability, the memory cells in the HRS have the ability to self-generate photocurrent signals. This allows for the transduction of the memristive state when exposed to light pulses, effectively addressing crosstalk issue during the optoelectrical readout operation. In contrast, conventional electrical readout methods can lead to undesirable sneak paths where incorrect data is outputted, as they bypass the intended target cell (Fig. 4a)^3,46^. The photoconductive waveform of a memory cell in the HRS hinges closely to the incident power, with its peak intensity decreasing proportionally with power density (Fig. 4b). The minimum detectable power density is measured at 0.06 μW/cm^2^, with a corresponding photocurrent of 1.68 pA. In comparison, the sensing current for the same cell in LRS is significantly lower at 1.16 pA and remains independent of the incident power (Fig. 4c). This indicates that the photocurrent on/off ratio in this particular cell is 1.44 at an incident power density of 0.06 μW/cm^2^. This ratio is further enhanced to 14.96 at an incident power density of 0.52 μW/cm^2^, thereby enabling the ultrasensitive sensing and recognition of the HRS.

As a proof-of-principle study, a 4 × 4 passive crossbar array comprising 16 MPT memory cells was constructed. The outer 12 cells were programmed to LRS while the middle 4 cells remained in HRS (Fig. 4d). Subsequently the resistance states of each cell in the array were distinguished using both electrical and optoelectrical reading methods. A readout bias of 0.5 V was applied to perform electrical reading. The sensed data, shown in Fig. 4e, revealed that the resistance values of the outer 12 cells ranged from 100 – 500 Ω, which is in consistent with the expected LRS values. However, when the middle HRS cell was selected, the current flowing through the neighbor cells became significant due to the absence of additional selective unit. Consequently, the sensed resistance value (800 ~ 1600 Ω) deviated significantly from the expected HRS values (<100 MΩ). This discrepancy leads to an erroneous interpretation of the actual data. Subsequently, the stored data of these MPT cells was retrieved by optoelectrical readout. Figure 4f shows the color map of the readout photocurrent under pulse light illumination (0.52 μW/cm^2^) with a reading bias of 0 V. The baby blue color for the inter 4 cells indicates a higher readout photocurrent, while the almost white color for the 12 peripheral cells suggests an ultralow sneak current (~1 pA). Thus, the true resistance states of these memory cells can be accurately identified. Notably, our optoelectronic reading is a non-destructive operation due to the 0 V readout bias loading, thereby effectively preserve the device endurance.

## Discussion

In summary, we present a MPT strategy that enables accurate and nondestructive readout in a semiconductor resistive memory array with a crossbar architecture. Each individual memory cell in this array exhibits resistive switching induced by conductive filament formation/rupture, as well as persistent photoconductance induced by charge trapping/de-trapping. Impressively, the fully connected conductive filament eliminates the photocurrent, which can be directly utilized as a signal for sensing the memory state of each cell. With this MPT feature, we successfully achieved accurate identification of the resistance states of the HRS or LRS cells in a 4 * 4 passive memory array. This innovative MPT strategy opens up a promising pathway for achieving crosstalk-free readout in advanced memory systems.

## Materials and methods

### Materials

The P3HT (average Mw 35,000–45,000) was purchased from Xi’an Polymer Light Technology Corp. Chloroform was purchased from Aladdin. ITO electrode was purchased from South China Science & Technology Company Limited. Silver particle for the preparation of electrodes were purchased from ZhongNuo Advanced Material (Beijing) Technology Co., Ltd. Ultrapure water (18.2 MΩ cm) was prepared by laboratory TANKPRO ultrapure water instrument.

### Device fabrication

A P3HT layer with uniformly distributed nanoholes was obtained by spin-coating the solution of P3HT in chloroform (5 mg/mL) on the precleaned ITO electrode at 3000 rpm for 40 s in damp air with the humidity of 30%–40%, followed by transferred to an oven at 70 °C for 30 min to remove the trace amount of chloroform. The Ag doped P3HT layer was fabricated by the identical process with the solution of AgNP:P3HT (diameter: 5 ~ 10 nm, concentration: 0.1% wt, 5 mg/mL) in chloroform. Subsequently, a 150 nm thick top Ag electrode was thermally evaporated on the P3HT layer through a shadow mask.

### Morphologies and surface potential characterizations

The atomic force microscopy (AFM) images were obtained by using a Dimension 3100 (Veeco, CA) in tapping mode in ambient conditions at room temperature. The cross-sectional STEM images as well as the EDS mapping were obtained with Tecnai F30. The investigation of charge trapping capacity was performed by AFM electrical technique (Bruker Dimension ICON, KPFM mode) in ambient conditions at room temperature, which integrates a light source module to realize the realtime measurement of surface potential under illumination.

### Memory and photoresponse measurements

All electrical and photoelectrical measurements were performed by a Keithley 4200-SCS semiconductor parameter analyzer in ambient conditions. Light pulses with different intensities come from high-power LED drivers (THORLABS, DC2200). The light source system is installed in the Keithley 4200 SCS shielding box to avoid interference from external light signals.

### Supplementary information

Supplementary material
