# Supplementary material for: A memristive-photoconductive transduction methodology for accurately nondestructive memory readout
Source: Light Sci Appl. 2024 Jul 23;13:175. doi: 10.1038/s41377-024-01519-w (PMC11266504; doi:10.1038/s41377-024-01519-w)
Supplement: Supplementary file 1 — Supplementary material [file 41377_2024_1519_MOESM1_ESM.doc]

Supplementary Information for

A Memristive-Photoconductive Transduction Methodology for Accurately Nondestructive Memory Readout

Zhe Zhou^1^, Yueyue Wu^1^, Keyuan Pan^1^, Duoyi Zhu^1^, Zifan Li^1^, Shiqi Yan^2^, Qian Xin^2^, Qiye Wang^1^, Xinkai Qian^1^, Fei Xiu^1^, Wei Huang^1,3^, Juqing Liu^1^*,

Correspondence to: *Corresponding author. Email: iamjqliu@njtech.edu.cn (J. L.);*

**Fig. S1.** (a) Schematic illustration of a porous P3HT film via the breath figure method. (b) The UV-visible absorption spectra of the P3HT film.

**Fig. S2.** (a) The endurance of the memory cell. (b) The retention performance for the memory cell in both HRS and LRS with a high ON/OFF ration >10^5^. (c) The statistic distribution of set voltage of 120 random selected memory cells. (d) The statistic distribution of reset voltage of 120 random selected memory cells.

**Fig. S3.** (a) The cyclic transient photoresponse of the pristine memory cell. (b) The cyclic transient photoresponse of the same memory cell (HRS) undergoing an integral set-reset process. (c) The amplified transient response shows that the relaxation time of pristine memory cell is ~ 0.93 s. (d) The amplified transient response shows an increase relaxation time (4.85 s) of the same memory cell (HRS) undergoing an integral set-reset process.

**Fig. S4.** (a) The surface potential images of pure P3HT film without and with illumination. (b) The surface potential images of Ag doped P3HT film without and with illumination. Scale bars: 250 nm.
